# Inhibition of cytoplasmic cap methylation identifies 5′ TOP mRNAs as recapping targets and reveals recapping sites downstream of native 5′ ends

**DOI:** 10.1093/nar/gkaa046

**Published:** 2020-01-30

**Authors:** Daniel del Valle Morales, Jackson B Trotman, Ralf Bundschuh, Daniel R Schoenberg

**Affiliations:** 1 Center for RNA Biology, Columbus, OH 43210, USA; 2 Department of Biological Chemistry and Pharmacology, Columbus, OH 43210, USA; 3 Molecular, Cellular and Developmental Biology Program, Columbus, OH 43210, USA; 4 Department of Physics, Columbus, OH 43210, USA; 5 Department of Chemistry and Biochemistry, Columbus, OH 43210, USA; 6 Division of Hematology, The Ohio State University, Columbus, OH 43210, USA

## Abstract

Cap homeostasis is the cyclical process of decapping and recapping that maintains the translation and stability of a subset of the transcriptome. Previous work showed levels of some recapping targets decline following transient expression of an inactive form of RNMT (ΔN-RNMT), likely due to degradation of mRNAs with improperly methylated caps. The current study examined transcriptome-wide changes following inhibition of cytoplasmic cap methylation. This identified mRNAs with 5′-terminal oligopyrimidine (TOP) sequences as the largest single class of recapping targets. Cap end mapping of several TOP mRNAs identified recapping events at native 5′ ends and downstream of the TOP sequence of EIF3K and EIF3D. This provides the first direct evidence for downstream recapping. Inhibition of cytoplasmic cap methylation was also associated with mRNA abundance increases for a number of transcription, splicing, and 3′ processing factors. Previous work suggested a role for alternative polyadenylation in target selection, but this proved not to be the case. However, inhibition of cytoplasmic cap methylation resulted in a shift of upstream polyadenylation sites to annotated 3′ ends. Together, these results solidify cap homeostasis as a fundamental process of gene expression control and show cytoplasmic recapping can impact regulatory elements present at the ends of mRNA molecules.

## INTRODUCTION

Loss of the mRNA 5′ cap is generally an irreversible step that leads to degradation by XRN1 (1). However, in 2009 we described a cytoplasmic complex of enzymes that is capable of restoring the cap onto RNAs with 5′-monophosphate ends (2), and others described the existence of capped ends within the body of mRNAs, downstream of the native (i.e. canonical) cap site (3). The cytoplasmic capping complex contains capping enzyme (RNGTT, referred to here as CE), a 5′-monophosphate kinase, and the heterodimer of cap methyltransferase (RNMT) with its activating subunit (RAMAC or RAM). This assembles on adapter protein NCK1, with CE bound to the third SH3 domain, the 5′ kinase bound to the second SH3 domain (4), and the RNMT:RAMAC heterodimer bound directly to CE (5). These findings are summarized in a recent review (6), where we also discuss the broader relationship of cytoplasmic capping to transcriptome and proteome complexity.

Although the biochemical steps in cytoplasmic capping are now established, less is known about characteristics of recapping targets and how these are selected. A recent proteomics analysis of the cytoplasmic CE interactome identified 66 interacting proteins, 52 of which are RNA-binding proteins (7). Based on those findings we proposed that target selectivity is determined by binding by one or more of these proteins. Their subsequent interaction with cytoplasmic CE then mediates assembly of the recapping complex on specific mRNPs.

Our previous work identified recapping targets by the appearance of uncapped transcripts when cytoplasmic capping was blocked by overexpression of an inactive form of CE (8). Fortuitously, many uncapped transcripts were fairly stable and could be identified by their *in vitro* susceptibility to digestion with XRN1 (8). However, this approach is limited to a metastable pool of uncapped transcripts and is dependent on biochemical separation of capped versus uncapped RNAs. Given the central role of NCK1 in both receptor tyrosine kinase signaling and in assembling the cytoplasmic capping complex, it is likely that the scope of recapping targets differs between cell types and in tissues. We therefore sought to develop a way of identifying recapped mRNAs that is broadly applicable and independent of cap status.

The approach we present here is based on the observation in (5) that cytoplasmic cap methylation could be inhibited by overexpression of a C-terminal portion of RNMT(121-476) carrying a mutation in the binding site for *S*-adenosylmethionine (termed ΔN-RNMT). Cells possess enzymes that degrade mRNAs with improperly methylated caps (9,10), and steady-state levels of several well-characterized recapping targets declined in cells expressing ΔN-RNMT whereas non-target mRNAs were unchanged. Here this approach is merged with quantitative RNA-Seq for transcriptome-wide identification of recapping targets. With this we identify mRNAs with 5′-terminal oligopyrimidine (TOP) sequences as the largest single group of recapping targets and provide the first direct evidence for recapping at both the native 5′ end and at downstream sites.

## MATERIALS AND METHODS

### Cloning of pcDNA3/TO-ΔN-RNMT plasmid

With pcDNA3-FLAG-RNMT 121–476 D203A (‘pcDNA3-ΔN-RNMT,’ (5); Addgene plasmid #112708) as template, Phusion Site-Directed Mutagenesis (Thermo Fisher F541) was used with forward primer JT130 (5′-CCTATCAGTGATAGAGATCTCCCTATCAGTGATAGAGATCTGGCTAACTAGAGAACCCAC-3′) and reverse primer JT127 (5′-GAGAGCTCTGCTTATATAGACCTCCCA-3′) to insert two copies of the tetracycline operator sequence (TetO_2_) between the CMV promoter TATA box and the transcription start site at the same location as in pcDNA4/TO (Thermo Fisher V102020). The sequence of the resulting plasmid, pcDNA3/TO-ΔN-RNMT, was verified by Sanger sequencing.

### Generation and culture of U2OS-TR/ΔN-RNMT stable cell line

Human U2OS osteosarcoma cells stably expressing the tetracycline repressor (U2OS-TR) were described previously in (2). To generate cells with tetracycline-inducible ΔN-RNMT stably integrated, U2OS-TR cells were transfected with pcDNA3/TO-ΔN-RNMT using Fugene 6 following the manufacturer's protocol. Cells were selected in medium containing 600 μg/ml G418 (Thermo Fisher 10131035), seeded at low density on new dishes, and individual colonies were isolated with cloning cylinders and expanded. Several clonal lines were tested by Western blotting for responsiveness to doxycycline induction of ΔN-RNMT, and the line with the greatest level of expression (#17) was chosen for this study. Cells were grown at 37°C and under 5% CO_2_ in McCoy's 5A medium (Thermo Fisher 116600) supplemented with tetracycline-free fetal bovine serum (Atlanta Biologicals S10350) to 10% (v/v).

### Immunofluorescence

U2OS-TR/ΔN-RNMT cells were seeded on glass coverslips and cultured for 25 h in medium with or without 1 μg/ml doxycycline before fixing with ice-cold methanol for 20 min. Coverslips were washed three times with PBS before blocking in IF Block Solution (PBS containing 1% (w/v) BSA and 0.05% (v/v) Triton X-100) at room temperature for 90 min. ΔN-RNMT was visualized by incubating at 4°C overnight with a 1:1000 dilution of mouse monoclonal anti-FLAG (Sigma F3165). Coverslips were washed three times for 5 min with IF Wash Buffer (PBS containing 0.5 mM MgCl_2_ and 0.05% (v/v) Triton X-100) and then incubated in the dark at room temperature for 60 min in IF Block Buffer containing a 1:1000 dilution of anti-mouse Alexa Fluor 680 (Thermo Fisher A21057) and 0.75 μg/ml DAPI. Coverslips were washed three times with IF Wash Buffer as before, mounted on glass microscope slides with ProLong Gold Antifade Mountant (Thermo Fisher P36930), and incubated in the dark at room temperature overnight to allow the mountant to cure. Images were acquired at room temperature with a Nikon Eclipse Ti-U inverted microscope fitted with a CFI Plan Apo VC 60× oil immersion objective and a Nikon DS-Qi1 monochrome digital camera. Images were analyzed using Nikon NIS-Elements AR 3.10 software. Specificity of the secondary antibody for the primary antibodies was confirmed by parallel preparation of control coverslips not treated with primary antibody.

### Western blotting

Cytoplasmic extracts were diluted to 1× Laemmli sample buffer, heated at 95°C for 5 min, and electrophoresed on Bio-Rad Mini-PROTEAN TGX SDS-PAGE gels at 150V in 1× Tris/glycine buffer containing 1% SDS (w/v). Proteins were then transferred to an Immobilon-FL PVDF membrane (Millipore Sigma IPFL00010) at 4°C and at 100V for 60 min in 1× Tris/glycine buffer containing 20% methanol (v/v) and 0.1% SDS (w/v). Membranes were blocked at room temperature in 3% BSA (w/v) in TBS for at least 30 min. Primary antibody staining was performed with rabbit anti-RNMT antibody (Proteintech 13743-1-AP, 1:500 dilution) or rabbit anti-EEF2 (One World Lab; 1:500 dilution) in 3% BSA (w/v) in TBS. Following three 10-min washes with TBS-T, membranes were incubated in the dark for 30 min in 3% BSA (w/v) in TBS containing a 1:10,000 dilution of anti-rabbit Alexa Fluor 680 (Thermo Fisher A21109). Membranes were washed with TBS-T as before, and Western blots were visualized on a Li-Cor Odyssey at 700 nm.

### Preparation of cytoplasmic RNA

3 × 10^6^ cells were split into a 10 cm dish and after 48h, 1 μg/ml of doxycycline was added. Twenty-four hours later, cells were rinsed once with ice cold phosphate buffered saline (PBS) and suspended in 1 ml of PBS with a cell scraper. The recovered cells were centrifuged at 2500 × g for 5 min at 4°C, washed once with 1 ml PBS and centrifuged again at 2500 × g for 5 min at 4°C. The pelleted cells were resuspended in 5 volumes of lysis buffer (20 mM Tris–HCl, pH 7.5, 150 mM NaCl, 5 mM MgCl_2_, 1 mM DTT, 0.2% NP-40, 80 U/ml RNaseOUT (Invitrogen)) and incubated on ice for 10 min. Nuclei were removed by centrifugation at 12 000 × g for 10 min at 4°C. The supernatant fraction was used for western blot analysis and for RNA isolation using Direct-zol RNA MiniPrep Kit (Zymo Research R2053) including an in-column DNAse I digestion. Purified RNA was eluted in RNase free water.

### Preparation and sequencing of QuantSeq REV libraries

Sequencing libraries were prepared from 2 μg of cytoplasmic RNA from uninduced or 24 h doxycycline-treated (induced) cells carrying the ΔN-RNMT transgene (*n* = 5 for each) or parental U2OS-TR cells (*n* = 3 for each) using the QuantSeq 3′ mRNA-Seq Library Prep Kit REV for Illumina (Lexogen) according to manufacturer's protocol. The final concentration of each library was determined using Qubit 2.0 Fluorometer (Invitrogen). Paired end 75 sequencing of libraries from ΔN-RNMT expressing cells was performed by Lexogen at the Vienna Biocenter Core Facility on an Illumina NextSeq 500. Paired end 150 sequencing of libraries from U2OS-TR cells was performed in the Genome Services Laboratory at Nationwide Children's Hospital, Columbus, OH, on an Illumina MiSeq.

### Quantitative RT-PCR

0.5 μg of cytoplasmic RNA was spiked with 1 fmol of CleanCap^®^ mCherry mRNA (Trilink L7023) and 0.5 μl of oligo(dT)_15_ primer (500 μg/ml) in a total volume of 10 μl. The mixture was incubated at 65°C for 5 min and immediately placed on ice. The mixture was brought to 20 μl with 4 μl of 25 mM MgCl_2_, 1 μl of 10 mM dNTPs, 4 μl of GoScript^®^ 5× Reaction Buffer and 1 μl of GoScript^®^ Reverse Transcriptase (Promega A2791). Reactions were placed in the thermocycler at 25°C for 5 min, 42°C for 1 h and 70°C for 15 min. The resulting cDNA was quantified by real time PCR in technical triplicate reactions containing 0.5 μM reverse and forward primer (Supplementary Table S4) and 1× SensiFAST SYBR No-ROX (Bioline, BIO-98005) with a Bio-Rad CFX Connect real-time PCR detection system. PCR was performed with the protocol of 95°C for 3 min, 40 cycles of (95°C for 10 s, 55°C for 30 s).

### Quantification and statistical analysis of RT-qPCR data

RT-qPCR data were analyzed using Bio-Rad CFX Maestro^®^ Software. Ct values were determined by regression mode. Fold change was determined by the ΔΔCq method corrected for the primer efficiency and normalized to the mCherry mRNA spike-in control and STRN4 as an endogenous non-target control. Values for uninduced (or 0h) samples were arbitrarily set to 1. Statistical analysis was performed with GraphPad Prism 6 and significance was determined by unpaired *t*-test, with results having *P* value <0.05 considered significant. For Figure 3B, GraphPad Prism 6 was used to plot the mean ± standard deviation of independent biological triplicates.

### 5′ end analysis

2 μg of cytoplasmic RNA spiked with 1 fmol of mCherry mRNA and 1 fmol uncapped Luciferase RNA (Promega) was used for gene specific 5′ end mapping using TeloPrime^®^ Full-Length cDNA Amplification Kit V1 (Lexogen) according to the manufacturer's protocol. The resulting cDNA was PCR amplified with MyTaq 2× mix (Bioline IO-21105) using gene specific reverse primers and the TeloPrime forward adapter primer. PCR samples were ethanol precipitated, separated on a 6% native PAGE gel, and bands were visualized using SYBR^®^ Gold Nucleic Acid Gel Stain (Thermo Fisher S11494). Bands of interest were excised from the gel and centrifuged through a 0.6 ml microtube for 1 min at 13 000 × g. The crushed gel slice was soaked in 3 volumes of nuclease free water and incubated overnight at room temperature with slight agitation. Eluted DNA was ethanol precipitated and sequenced using gene specific reverse primers at the Genomics Shared Resource at The Ohio State University. The doublet bands of EIF3K and EIF3D were extracted as described above. The recovered DNA was incubated with MyTaq 2× mix (Bioline) at 70°C for 20 min to add overhanging A residues and purified using DNA Clean and Concentrator-5 (Zymo). These were then ligated into pGEM^®^-T Easy Vector System (Promega A1360) for 1 h at room temperature using a 3:1 insert to vector ratio and T4 DNA ligase (Promega M1801) and transformed into Stellar Competent Cells (Clontech) cells following the manufacturer's protocol. Transformed cells were plated on LB/ampicillin plates and incubated overnight at 37°C. Individual colonies were grown in liquid medium (LB/ampicillin), and plasmid DNA was recovered using NucleoSpin Plasmid kit (Clontech). The purified plasmids were sequenced at the Genomics Shared Resource at The Ohio State University using T7 promoter forward primer, and capped ends were identified as the sequence immediately adjacent to the TeloPrime adaptor.

### Bioinformatics

Data reduction was performed using the REV Human (GRCh38) Lexogen QuantSeq 2.2.3 pipeline from the Lexogen Blue Bee platform. Files were filtered for base mean read count >20 across all samples (12,134 genes), and differential gene expression profiling was performed using DESeq2 (11) on Galaxy (12). Gene groups were identified by their statistical overrepresentation using default settings in the PANTHER (Protein ANalysis THrough Evolutionary Relationships) Classification System (13,14), version 14.0 released 2018-12-03. The Annotation Data Set was set to PANTHER Protein Class and analysis was performed using Fisher's Exact Test with False Discovery Rate correction. To identify genes with changes in 3′-UTR usage, reads in the terminal 50 nucleotides of each gene were counted separately from reads throughout the entire gene body for each hGRC38 RefSeq gene using featureCounts (15). Read counts in the terminal 50 nucleotides were provided as ‘Ribo-Seq’ data to RiboDiff (16) while total transcript length read counts were provided as ‘RNA-Seq’ such that RiboDiff would identify genes with changes in the ratio between read counts at the annotated 3′UTR end and read counts across all possible 3′UTR ends as a function of ΔN-RNMT induction. Genes were considered significant if the multiple testing corrected *P*-value for a change in the ratio reported by RiboDiff was <0.05.

## RESULTS

### An inducible system for studying the impact of inhibiting cytoplasmic cap methylation

**Figure 1.**
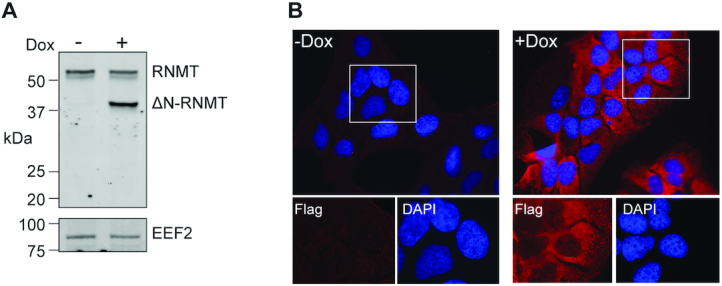
An inducible cell system to inactivate cytoplasmic cap methylation. A line of tetracycline-inducible U2OS cells was transfected with a plasmid bearing a tetracycline-regulated transgene expressing ΔN-RNMT. Clonal lines were selected by growth in medium containing G418 and analyzed for potential leakiness and inducible expression. One clonal line was selected for use in this study. (**A**) Cells carrying the ΔN-RNMT transgene were cultured for 24 h in the absence (–) or presence (+) of doxycycline (Dox), and cytoplasmic extracts were analyzed by western blotting with anti-RNMT antibody. The lower panel shows a portion of the same blot probed with antibody to EEF2 used in (7). (**B**) The same cell line grown on coverslips was again cultured for 24 h in the absence or presence of doxycycline, then fixed and stained with DAPI and antibody to the FLAG tag on ΔN-RNMT. The areas in white boxes are shown enlarged at the bottom of the figure.

Tetracycline-inducible U2OS cells were transfected with a plasmid expressing ΔN-RNMT under tetracycline operator control, and clonal lines were characterized for tightness of expression and degree of inducibility. The western blot in Figure 1A shows one such line where ΔN-RNMT was only expressed in doxycycline-treated cells, and its induction had no impact on endogenous RNMT. ΔN-RNMT lacks the N-terminal nuclear localization sequences of the native protein, and addition of the HIV Rev NES restricts its distribution to the cytoplasm. Immunofluorescence shows no evidence for ΔN-RNMT expression in the absence of doxycycline treatment, and following doxycycline addition, the induced protein is restricted to the cytoplasm (Figure 1B). Thus, it is unlikely that changes observed upon induction of ΔN-RNMT result from inhibition of nuclear cap methylation.

### Targeting cap methylation to identify recapping targets

**Figure 2.**
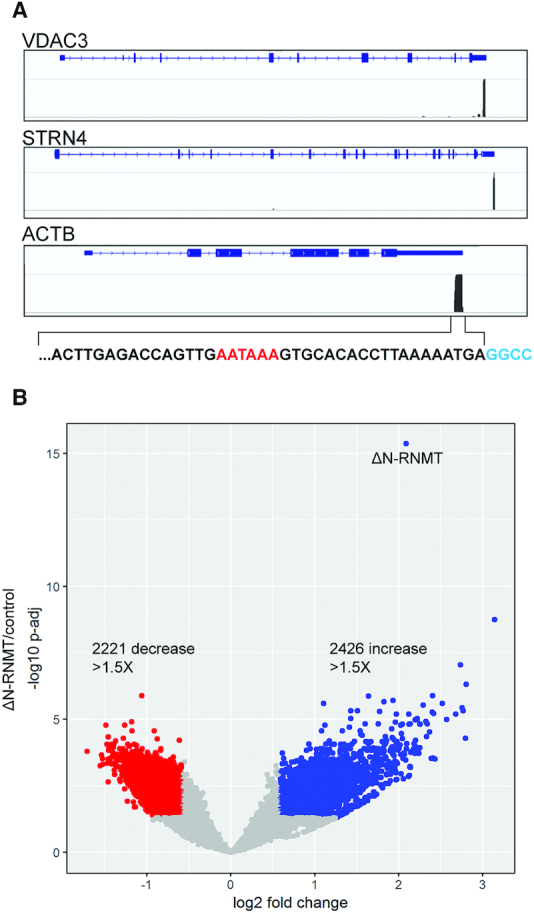
Positioning of 75 bp sequence tags and quantitative changes following inhibition of cytoplasmic cap methylation. (**A**) The genome locations of the QuantSeq REV sequence tags are shown for one cytoplasmic capping target originally identified in (8) (VDAC3) and two non-target mRNA (STRN4, ACTB). A portion of the latter 75 bp sequence tag is shown in black with the AAUAAA poly(A) signal highlighted in red. (**B**) The QuantSeq REV data from Supplementary Table S2 are displayed by volcano plot, with transcripts that decrease with ΔN-RNMT shown in red and those that increase shown on blue.

Quantitative changes in transcript levels upon ΔN-RNMT expression were determined using the QuantSeq^®^ 3′ mRNA RNA-Seq REV (Lexogen) system. Rather than sequencing the entire transcriptome, this approach generates libraries of 3′-UTR sequence tags adjacent to the poly(A) tail. Libraries were prepared from cytoplasmic RNA of 5 biological replicates of control and ΔN-RNMT expressing cells. The reproducibility of this approach is shown in Supplementary Figure S1A, and metagene analysis confirmed sequence tags are concentrated at the mRNA 3′ end (Supplementary Figure S1B). Additional confirmation was obtained by directly visualizing the locations of sequence tags on randomly selected transcripts. Examples of this are shown in Figure 2A for one recapped mRNA (VDAC3) and two controls (STRN4, ACTB). Sequence data beneath the ACTB shows the tag corresponds to the 75 nt immediately upstream of the poly(A) addition site. To control for potential off-target effects of doxycycline (17), we also prepared and sequenced libraries from parental U2OS-TR cells (Supplementary Figure S1C). Only a single non-coding RNA declined significantly with doxycycline treatment, thus indicating any changes observed are specific to ΔN-RNMT.

### TOP mRNAs are susceptible to disruption of cytoplasmic cap methylation

5606 mRNAs undergo some degree of change following induction of ΔN-RNMT (Supplementary Table S1), with approximately equal numbers decreasing and increasing >1.5-fold (Figure 2B). Because our experimental paradigm was based on the loss of mRNAs with improperly methylated caps, we first focused on transcripts that decline with ΔN-RNMT induction. mRNAs were classified into distinct groups using Protein Analysis Through Evolutionary Relationships (PANTHER, (13,14)), which integrates gene ontology with function. Using a false discovery rate of <0.05, this grouped the downregulated transcripts into 4 main categories: ribosomal proteins, membrane traffic proteins, transferases, and RNA-binding proteins (Table 1).

**Table 1.** PANTHER analysis of mRNAs downregulated in ΔN-RNMT expressing cells

PANTHER protein class	REFLIST (20996)	# Down with ΔN-RNMT	Expected	Fold enrichment	Raw *P*-value	FDR
Ribosomal protein	160	39	15.93	2.45	4.45 × 10^−6^	3.19 × 10^−4^
Membrane traffic protein	280	51	27.89	1.83	2.13 × 10^−4^	7.63 × 10^−3^
Transferase	866	129	86.25	1.50	2.79 × 10^−5^	1.50 × 10^−3^
RNA binding protein	636	92	63.34	1.45	1.01 × 10^−3^	3.11 × 10^−2^

The downregulated genes in Supplementary Table S2 were analyzed by PANTHER (Protein Analysis Through Evolutionary Relationships) using Fisher's Exact Test with False Discovery Rate correction. The data indicate the fold enrichment over expected representation of gene families.

**Figure 3.**
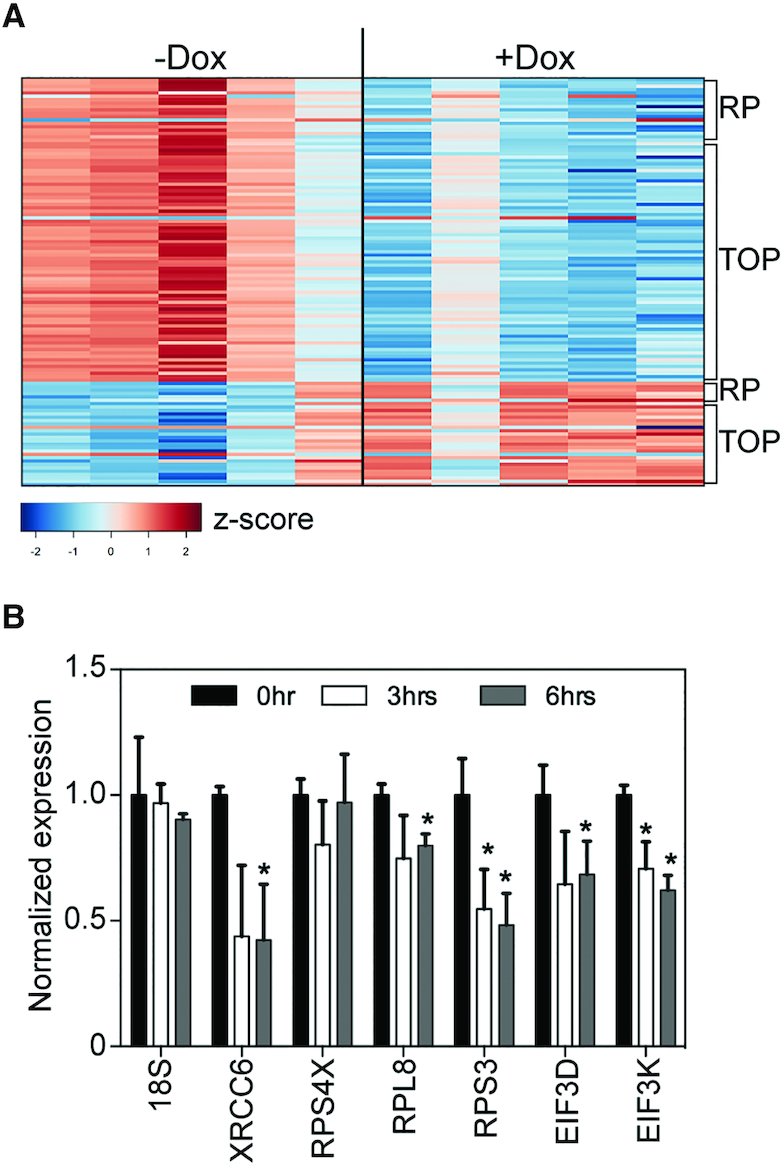
Differential expression of TOP mRNAs in response to inhibition of cytoplasmic cap methylation. (**A**) A heat map is shown for TOP mRNAs identified as changing in the five replicate libraries prepared after culturing in medium without (–Dox) or with (+Dox) doxycycline to induce ΔN-RNMT expression. Ribosomal protein mRNAs are indicated on the right-hand side as RP, and non-ribosomal protein TOP mRNAs are indicated as TOP. The scale indicates *z*-scores for each determination. (**B**) Cytoplasmic RNA was recovered from cells carrying ΔN-RNMT prior to addition of doxycycline to the medium and at 3 and 6 h after addition. Each preparation was spiked with an equal amount of mCherry mRNA and analyzed by RT-qPCR for 18S rRNA and XRCC6, RPS4X, RPL8, RPS3, EIF3D and EIF3K mRNA. The results were normalized to mCherry and are shown as the mean ± standard deviation (*n* = 3). *P < 0.05 by unpaired two-tailed *t*-test.

Ribosomal protein transcripts are among the most abundant mRNAs, but they are also part of a larger group of mRNAs characterized by the presence of a 5′ terminal oligopyrimidine (TOP) sequence. TOP mRNAs encode proteins involved in translation initiation, elongation, and termination. The TOP sequence is immediately adjacent to the cap (18), and translation of TOP mRNAs is regulated by binding of La-related protein 1 (LARP1) to the cap and TOP sequence (19–21). Using recovery with LARP1 as a metric for their identification, Gentilella *et al.* (22) classified 310 transcripts as TOP mRNAs. One hundred sixteen of these are in our pool of downregulated transcripts (Supplementary Table S2) and constitute the largest single grouping of cytoplasmic capping targets (*P* = 2.4 × 10^−12^ by two-tailed Fisher's exact test). A number of TOP mRNAs were also among those that increased in ΔN-RNMT expressing cells, and these are shown together with the downregulated mRNAs in Figure 3A.

These findings were confirmed by RT-qPCR using cytoplasmic RNA recovered from triplicate cultures of untreated cells or cells in which ΔN-RNMT was induced for 3 or 6 h (Figure 3B). This analysis included three ribosomal proteins (RPS4X, RPL8, RPS3), two non-ribosomal TOP mRNAs (EIF3K and EIF3D), one non-TOP mRNA that is also down regulated (XRCC6), with 18S rRNA used as a negative control. ΔN-RNMT had no impact on 18S rRNA, and RPS3, RPL8, EIF3K, EIF3D and XRCC6 mRNAs were all less abundant in ΔN-RNMT expressing cells. There was some evidence for a decrease in RPS4X mRNA levels at 3 h, but the results were not statistically significant. The products of TOP mRNAs, such as ribosomal proteins, are generally stable. We therefore did not expect to see significant change in steady-state levels of representative proteins. This proved to be the case for RPS4X, RPS3 and EIF3D, however there was evidence for modest decreases in RPL8 and EIF3K (Supplementary Figure S2).

### Recapping occurs at the native 5′ end and downstream of the TOP sequence

**Figure 4.**
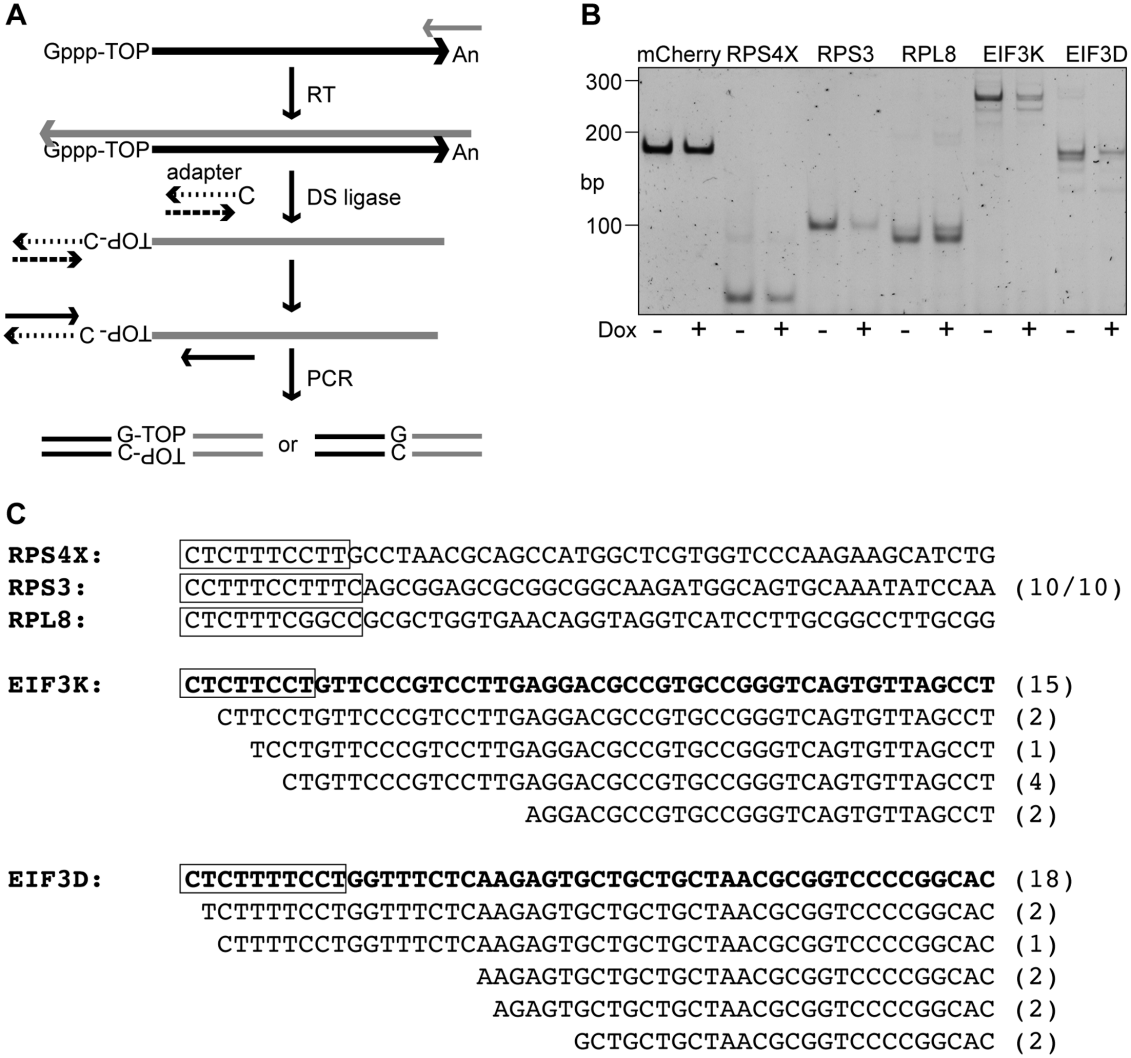
Characterization of TOP mRNA 5′ ends. (**A**) A diagram is shown of the approach to mapping TOP mRNA 5′ ends using the Lexogen TeloPrime^®^ system. A double-stranded DNA adapter is ligated onto the cDNA using a double-strand DNA ligase provided with the kit. 5′ ends are then PCR amplified using primers downstream of the TOP sequence and a primer to the ligated adapter. The validity of this approach for identifying capped ends has been validated using capped mCherry as a positive control and 5′ triphosphate firefly luciferase mRNA and 18S rRNA as negative controls. (**B**) Cytoplasmic RNA from uninduced (–) and 24 h doxycycline-treated cells (+) was spiked with capped mCherry mRNA, reverse transcribed, ligated to the double-strand adapter and PCR amplified and separated on a 6% native polyacrylamide gel that was then stained with SYBR Gold. The cycle numbers for each gene were: RPS3, 15 cycles; RPS4X and RPL8, 18 cycles; EIF3K, 19 cycles; mCherry and EIF3D, 22 cycles. (**C**) The PCR amplified products from uninduced cells were recovered from the gel. The single RPS4X, RPS3 and RPL8 bands were sequenced directly. The first 50 bp of each mRNA are shown and these correspond to their respective RefSeq sequences. RPS3 and the double bands for EIF3K and EIF3D were cloned, and plasmids were sequenced from 10 clones of RPS3, 24 clones of EIF3K and 27 clones of EIF3D (parentheses). The double bands for EIF3K and EIF3D were excised in a single gel piece, the recovered DNA was cloned, and plasmids were sequenced from 24 and 27 independent clones of EIF3K and EIF3D, respectively. Capped ends were identified as the sequence appended immediately adjacent to the TeloPrime adapter sequence. TOP sequences are indicated with a box, the RefSeq sequences for the first 50 nucleotides of EIF3K and EIF3D are in bold, and the respective 5′ end truncations are showed with respect to this sequence.

There is evidence from capped analysis of gene expression (CAGE) for capped ends downstream of native 5′ ends (23). We previously identified recapping targets by the appearance of their uncapped forms in cells expressing a dominant negative form of cytoplasmic CE (8), and the 5′ ends of a number of these map to positions of downstream CAGE tags (24,25). Based on this we wondered if recapping might bypass the TOP sequence and with it, regulation by LARP1. To address this, we looked at capped ends on RPS3, RPS4X, RPL8, EEF1D and EIF3D mRNA using the Lexogen TeloPrime^®^ system. TeloPrime is designed to generate full length cDNAs for sequencing using a proprietary ligase to covalently append a double stranded adapter with an overhanging C residue onto the cDNA 3′ end (Figure 4A). If recapping occurs at the native 5′ end, PCR with primers to the ligated adapter and the gene of interest will yield a single product. The same holds true for mRNAs that also undergo downstream recapping, except these would generate two or more PCR products.

PCR products for RPS4X, RPS3, RPL8, EIF3K, EIF3D and the mCherry spike-in control are shown in Figure 4B. Differences in ligation efficiency impact approaches to quantifying changes in 5′ ends, particularly for transcripts with multiple 5′ ends (see below). We addressed this by limiting cycle number to the minimum needed to visualize products. RPS4X, RPS3 and RPL8 each yielded a single PCR product of the size expected for mRNAs starting at the native cap site, and in agreement with RT-qPCR data band intensity was lower for RPS4X and RPS3 in samples from ΔN-RNMT expressing cells. A slightly larger band was observed with ΔN-RNMT for RPL8, but this was not examined further. EIF3K and EIF3D each generated bands expected for native capped ends that declined with ΔN-RNMT, as well as a faster migrating band consistent with downstream recapping products.

Direct Sanger sequencing of the RPS4X, RPS3 and RPL8 PCR products indicated the TeloPrime adapter was appended to the RefSeq 5′ ends of their corresponding mRNAs. This also matched sequences determined by nanoCAGE (26) (Figure 4C). As an additional control the RPS3 PCR product was cloned, and sequencing of 10 independent colonies yielded the same result. Thus, recapping occurred at the native 5′ end of each of these ribosomal mRNAs. Because the doublet bands for EIF3K and EIF3D could not be cleanly separated they were excised in a single gel piece and cloned. 24 clones of EIF3K and 27 clones of EIF3D were then sequenced, the results of which are shown in the bottom of Figure 4C. Like RPS3, 15 clones of EIF3K and 18 clones of EIF3D had adapter ligated at the 5′ ends of their corresponding mRNAs, indicating that these undergo recapping at their native 5′ ends. The remaining clones showed adapters ligated onto 5′ end truncations, indicative of downstream recapping. Several of these retained most of the TOP sequence; however, this was missing from a number of clones, most notably of EIF3D. In addition to its ramifications for regulation (see Discussion) this finding is the first direct proof for cytoplasmic capping mediating the addition of a downstream cap.

### The upregulated genes are enriched for DNA binding proteins, transcription factors and RNA processing proteins

As noted above, a number of mRNAs somewhat unexpectedly increased following induction of ΔN-RNMT (Figure 2B and Supplementary Table S1). When analyzed by PANTHER, the upregulated genes fell into related categories associated primarily with transcription and RNA processing (Table 2). This was unlikely due to cell stress as there was no evidence for increased eIF2α phosphorylation or for changes in translation as determined by pulse labeling with puromycin (Supplementary Figure S3). Compensatory changes in gene expression have been described (27), and we suspect these changes may be a compensatory response to the decline in a substantial number of transcripts or a widespread, secondary response to decreased levels of protein(s) encoded by one or more recapping targets.

**Table 2.** PANTHER analysis of mRNAs upregulated in ΔN-RNMT expressing cells

PANTHER protein class	REFLIST (20996)	#up with ΔN-RNMT	Expected	Fold enrichment	Raw *P*-value	FDR
Ubiquitin-protein ligase	98	27	10.88	2.48	1.19 × 10^−4^	2.13 × 10^−3^
Ligase	252	54	27.98	1.93	3.19 × 10^−5^	7.61 × 10^−4^
Chromatin/chromatin-binding protein	122	30	13.54	2.21	3.16 × 10^−4^	4.85 × 10^−3^
DNA binding protein	469	89	52.07	1.71	8.40 × 10^−6^	3.61 × 10^−4^
Nucleic acid binding	1599	279	177.52	1.57	4.04 × 10^−12^	8.69 × 10^−10^
mRNA splicing factor	109	25	12.10	2.07	2.08 × 10^−3^	2.23 × 10^−2^
mRNA processing factor	150	38	16.65	2.28	2.33 × 10^−5^	6.25 × 10^−4^
RNA binding protein	636	111	70.61	1.57	2.26 × 10^−5^	6.93 × 10^−4^
Kinase modulator	137	31	15.21	2.04	7.00 × 10^−4^	1.00 × 10^−2^
Transcription cofactor	167	35	18.54	1.89	1.29 × 10^−3^	1.63 × 10^−2^
Transcription factor	1156	191	128.34	1.49	5.02 × 10^−7^	5.39 × 10^−5^
Kinase	368	65	40.86	1.59	8.65 × 10^−4^	1.16 × 10^−2^
Transferase	866	144	96.14	1.50	1.03 × 10^−5^	3.69 × 10^−4^

The upregulated genes in Supplementary Table S2 were analyzed by PANTHER as in Table 1 using Fisher's Exact Test with False Discovery Rate correction.

### Inhibiting cytoplasmic cap methylation impacts alternative polyadenylation

**Figure 5.**
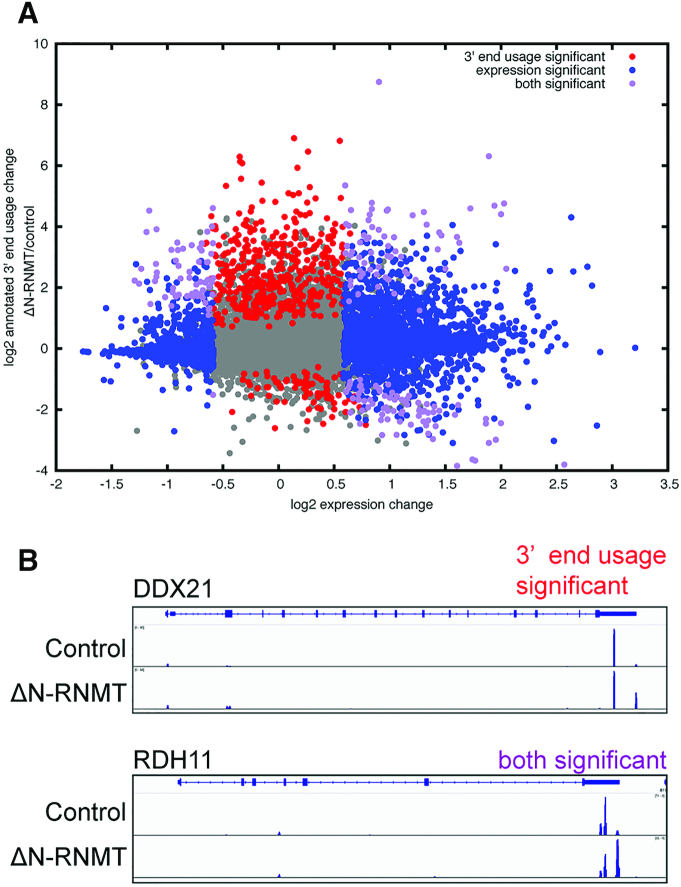
Changes in 3′ end usage associated with inhibition of cytoplasmic cap methylation. (**A**) RiboDiff was used to compare locations of QuantSeq REV 3′ end sequence tags from control and ΔN-RNMT expressing cells against the reference genome. Shown are for each gene the change in 3′ end usage and the change in overall expression. Significant (and beyond 1.5-fold) changes in transcripts from Supplementary Table S1 are shown in blue, transcripts for which there were only significant changes in annotated 3′ end use are shown in red, and transcripts that had significant changes in both quantity and annotated 3′ end usage are shown in purple. Genes without significant changes are shown in gray. (**B**) Examples of one gene from each group having significant changes in 3′ end usage are shown.

Bioinformatics performed in (28) classified the initial group of recapping targets (8) as coming from multi-UTR genes. Although alternative polyadenylation impacts much of the transcriptome (29), that finding suggested a possible link to target identification. To address this, we quantified alternative 3′ end formation via the fraction of 3′ end sequence tags in a gene aligning to the annotated 3′ processing site of the gene for all RefSeq genes in the GRCh38 reference genome. RiboDiff was used to compare these fractions from control and ΔN-RNMT expressing cells, reasoning that our quantity of interest was a ratio just like the ratio of ribosome protected fragments to RNA-Seq reads in ribosome profiling. Genes with significant changes are shown graphically in Figure 5A (and listed in Supplementary Table S3), with changes in 3′ ends superimposed on the quantitative data from Figure 2B and Supplementary Table S1. The absence of significant overlap between altered 3′ end usage and altered transcript levels (purple dots) indicates alternative polyadenylation is not a major determinant of target selection. In fact, these doubly significant genes were more prominent in the population of transcripts that increased with ΔN-RNMT and thus are likely not targets of cytoplasmic recapping. This analysis also identified transcripts that underwent changes in 3′ ends but not their steady-state level (red dots). No discernible ontological groupings were revealed by PANTHER analysis of these two datasets, suggesting changes in their 3′ ends are unlikely to be related to biochemical pathways of their encoded proteins.

The overall pattern in Figure 5A is one in which ΔN-RNMT expression resulted in increased use of annotated 3′ processing sites. The corollary to this that, in general, the mRNAs expressed in U2OS cells have shortened 3′-UTRs as a consequence of upstream polyadenylation. This is a common feature of cancer cell lines (30), but the degree of change observed here was unexpected, and we suspect this effect is secondary to changes in levels of 3′ processing factors as a consequence of inhibiting cytoplasmic cap methylation, notably PABPN1, whose mRNA and protein levels are increased 2- and 1.7-fold, respectively (Supplementary Table S1, Supplementary Figure S4), in ΔN-RNMT expressing cells. PABPN1 plays a major role in selection of alternative polyadenylation sites (31), and its increase here provides a likely explanation for the observed shift toward distal 3′ processing sites. A general shift in 3′ processing sites was confirmed by direct examination of changes in sequence tags as a function of ΔN-RNMT expression. Figure 5B shows examples of 2 randomly selected genes from each of the categories identified in Figure 5A. Some genes retained a population of transcripts with shortened 3′ UTRs whereas others showed a complete shift to annotated 3′ ends.

## DISCUSSION

Our initial identification of cytoplasmic capping targets was based on the appearance of uncapped transcripts following overexpression of a dominant negative form of CE. This required a number of assumptions, not the least of which was that uncapped transcripts were sufficiently stable to be detected (8). In addition, target identification required downstream biochemical separation of capped and uncapped RNAs. The current study used a different approach that circumvents these issues. It is based on the observation in (5) that the steady-state levels of several known recapping targets were lower in cells expressing an inactive, cytoplasmically-restricted form of RNMT, termed ΔN-RNMT. Because cap surveillance enzymes degrade mRNAs with improperly methylated caps, we hypothesized that target identification could be simplified by combining inhibition of cytoplasmic cap methylation with RNA-Seq. The identification of ribosomal protein mRNAs as recapping targets (Table 1) led us to examine the larger question of whether TOP mRNAs are targets of cytoplasmic capping. While there is some ambiguity as to the exact number of TOP mRNAs, we based the classification used here on the 310 transcripts identified by recovery with LARP1 (22). Using this criterion, our analysis identified 116 TOP mRNAs as cytoplasmic capping targets (Figure 3A, Supplementary Table S2), and this represents the largest single group of mRNAs identified to date that are regulated by cap homeostasis. Since TOP mRNAs are localized early to stress granules and P bodies (32), their identification here may be relevant to earlier results showing that inhibition of cytoplasmic capping reduced recovery from a brief arsenite stress (2). LARP1 is involved in tethering TOP mRNAs to stress granules, and since LARP1 binds both the cap and TOP sequence (19–21), cytoplasmic recapping may be needed to maintain TOP mRNAs in a functional state for translation once stress is removed.

A major unanswered question was whether mRNA recapping is restricted to the native cap site or if this also occurs downstream. Results in (8) showed mRNAs with uncapped ends corresponding to the native 5′ end and downstream sites accumulated in cells expressing a dominant negative form of cytoplasmic capping enzyme. Approximately 25% of CAGE tags map downstream within spliced exons (23), and using the same approach, Kiss *et al.* (24) and Berger *et al.* (25) mapped uncapped ends of a number of cytoplasmic capping targets to the vicinity of downstream CAGE tags. Since uncapped ends represent a precursor state, this approach can only infer their identity as recapping sites.

This question was addressed by tagging capped ends using an approach that adds a double-stranded primer onto first-strand cDNA immediately next to the site of a cap, in a cap-dependent manner, (Figure 4A) followed by PCR with a primer matching the adapter and gene specific primers for several TOP mRNAs. RPS4X, RPS3 and RPL8 each generated a single product (Figure 4B), and direct sequencing identified each of these products as retaining their native 5′ end (Figure 4C). Thus, as proposed in (8), cap homeostasis functions to maintain the capped ends of these ribosomal protein mRNAs. The same approach generated 2 closely spaced bands from EIF3K and EIF3D that were identified by cloning and sequencing. While the majority of sequences matched the native 5′ ends of their corresponding mRNAs, a number of these, most notably for EIF3D, mapped downstream of the TOP sequence. The corresponding transcripts retain the start site and coding sequence, and would presumably constitute a form of EIF3D that is immune to regulation by LARP1, and by extension, mTOR. eIF3D is a cap-binding protein that functions independently of eIF4F to affect the translation of a subset of the transcriptome (33,34) and mRNAs with 5′-UTR m^6^A (35). As such the existence of a LARP1-independent form of EIF3D mRNA may have implications for the regulation of non-canonical translation initiation, such as that using eIF3D and DAP5 (36).

A question that then follows is why recapped ends of TOP mRNAs are limited to the native cap site or just downstream of this. A possible answer may lie in the finding that the 5′ ends of TOP mRNAs are highly structured (37), and such regions limit XRN1 processivity (38). PANTHER analysis also yielded some insights into the unanticipated increase in a significant number of mRNAs. These fell into a number of related categories including DNA-binding proteins, transcription factors/co-activators, and proteins involved in pre-mRNA splicing, together suggestive of a ramping up of gene expression. Similar to results in (27) we suspect the overall increase in gene expression machinery may be compensation for the decreased levels of recapping targets and their encoded proteins.

Finally, the global increased use of annotated poly(A) sites following induction of ΔN-RNMT in U2OS cells was unexpected. Shortened 3′-UTRs are a common feature of cancer and cancer cell line transcriptomes (30), and in retrospect it is not surprising that in uninduced cells many of the 3′ end sequence tags are upstream of 3′ ends in the reference genome. The broad shift to distal (i.e. annotated) 3′ ends following ΔN-RNMT indicates upstream alternative polyadenylation was either directly or indirectly overcome by inhibiting cytoplasmic cap methylation. Given the breadth of the effect and the fact that the affected transcripts did not fall into distinct ontological groupings, we suspect changes in poly(A) site utilization are secondary to changes in one or more of the core 3′ processing components. ΔN-RNMT expression had no impact on mRNAs for CFIm or CFIIm; however, PABPN1 mRNA is almost 2-fold higher and PABPN1 is 1.7-fold higher in ΔN-RNMT expressing cells. PABPN1 is a suppressor of alternative polyadenylation (31), and reduced levels are associated with increased use of proximal poly(A). The increase in PABPN1 associated with ΔN-RNMT expression is therefore consistent with the global shift to distal cleavage and polyadenylation sites observed here.

In summary, the current study succeeded in identifying new cytoplasmic capping targets by their decrease following inhibition of cytoplasmic cap methylation. The most notable of these were the TOP mRNAs. Perhaps of greater importance, results presented here provide the first direct proof for cytoplasmic capping downstream of the native cap site. This finding opens the door for future work characterizing the relationship of cytoplasmic capping to transcriptome and proteome complexity.

## DATA AVAILABILITY

QuantSeq data are deposited in the Short Read Archive under BioProject ID PRJNA547607 and in GEO under accession GSE142848. QuantSeq data are also uploaded to the UCSC genome browser at https://genome.ucsc.edu/s/rbund/DeltaN%2DRNMT%2Didentifies%2DTOP%2DRNAs. We confirm transcriptome-wide analyses presented here have been validated by RT-qPCR.

## Supplementary Material

gkaa046_Supplemental_Files

## Notes

Present address: Jackson B. Trotman, Department of Pharmacology and Lineberger Cancer Center, University of North Carolina, Chapel Hill, NC 27599, USA.
