# Supplementary material for: Inhibition of cytoplasmic cap methylation identifies 5′ TOP mRNAs as recapping targets and reveals recapping sites downstream of native 5′ ends
Source: Nucleic Acids Res. 2020 Jan 30;48(7):3806–15. doi: 10.1093/nar/gkaa046 (PMC7144985; doi:10.1093/nar/gkaa046)
Supplement: gkaa046_Supplemental_Files [file gkaa046_supplemental_files.zip › Supplementary Material fixed for proof.pdf]

Supplementary Material

Del Valle Morales, D., Trotman, J.B., Bundschuh, R. and Schoenberg, D.R.
Inhibition of cytoplasmic cap methylation identifies TOP mRNAs as recapping targets and reveals recapping sites downstream of native 5' ends

Figure S1 (Related to Figure 2). Validation of QuantSeq REV libraries

Figure S2 (Related to Figure 3). Impact of ΔN-RNMT on proteins expressed from TOP mRNAs

Figure S3 (Related to Table 2). ΔN-RNMT induction does not impact the integrated stress response or global translation

Figure S4 (Related to Figure 5). PABPN1 is elevated in ΔN-RNMT expressing cells

Table S1. List of transcripts filtered for low read count and padj <.05

Table S2. List of TOP mRNAs that are downregulated in ΔN-RNMT expressing cells

Table S3. Analysis of 3' end changes as a function of ΔN-RNMT expression

Table S4. List of primers use for RT-qPCR and 5' end mapping

A

	-Dox1	-Dox2	-Dox3	-Dox4	-Dox5	+Dox1	+Dox2	+Dox3	+Dox4	+Dox5
-Dox1	1	1	0.95	1	0.96	0.95	0.98	0.95	0.95	0.91
-Dox2	1	1	0.96	1	0.95	0.94	0.98	0.94	0.94	0.9
-Dox3	0.95	0.96	1	0.95	0.87	0.87	0.91	0.86	0.89	0.85
-Dox4	1	1	0.95	1	0.97	0.96	0.99	0.96	0.96	0.93
-Dox5	0.96	0.95	0.87	0.97	1	0.99	0.99	0.99	0.98	0.96
+Dox1	0.95	0.94	0.87	0.96	0.99	1	0.98	1	0.99	0.95
+Dox2	0.98	0.98	0.91	0.99	0.99	0.98	1	0.98	0.98	0.94
+Dox3	0.95	0.94	0.86	0.96	0.99	1	0.98	1	0.99	0.96
+Dox4	0.95	0.94	0.89	0.96	0.98	0.99	0.98	0.99	1	0.97
+Dox5	0.91	0.9	0.85	0.93	0.96	0.95	0.94	0.96	0.97	1

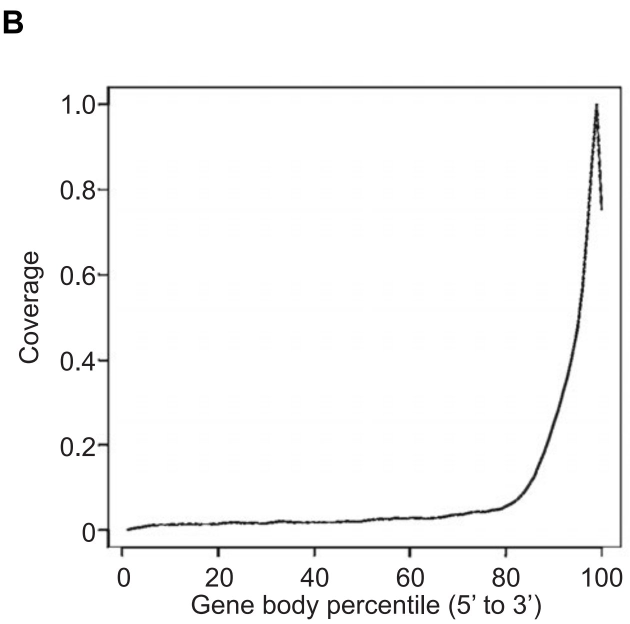

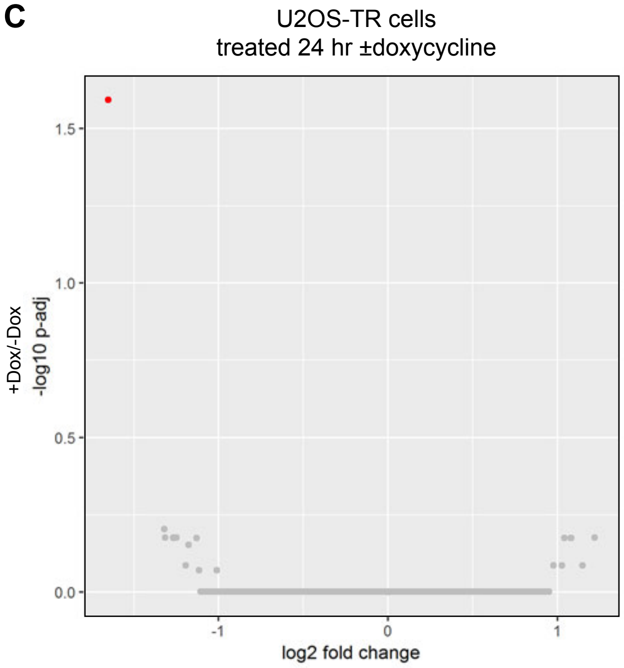

Figure S1 (Related to Figure 2)

Validation of QuantSeq REV libaries. A. Correlation coefficients are shown for scatter plots comparing each of the 5 replicate libraries for uninduced (-Dox) and ΔN-RNMT (+Dox) expressing cells. **B.** Metagene analysis was used to determine the distribution of 75 bp sequence tags after normalizing for gene length of sequenced transcripts. **C.** Volcano plot of QuantSeq REV libraries prepared from triplicate cultures of parental tet-inducible U2OS cells lacking the ΔN-RNMT transgene. PE150 sequencing was performed on an Illumina MiSeq in the Genomics Services Laboratory at Nationwide Children's Hospital, and differential gene expression was determined using DEseq2 on the Galaxy platform.

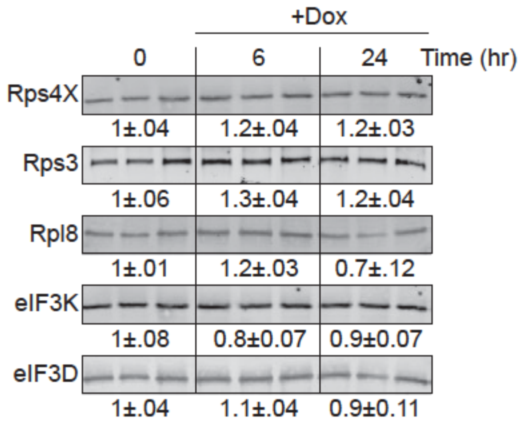

Figure S2 (Related to Figure 3)

Impact of ΔN-RNMT on proteins expressed from TOP mRNAs. Cytoplasmic extracts were prepared from triplicate cultures of U2OS cells carrying the inducible ΔN-RNMT transgene that were incubated without (time 0) or with 1 µg/ml doxycycline (+Dox) for 6 and 24 hr. Individual protein products of the TOP mRNAs from Figure 4 were analyzed by Western blotting with antibodies to Rps4X (Abclonal, A6730), Rps3 (Abclonal A2533), Rpl8 (Abclonal A10042), eIF3K (Abclonal A9969) and eIF3D (Abclonal A5947). The numerical values represent the mean ± standard deviation normalized to Ponceau S staining and values at time 0.

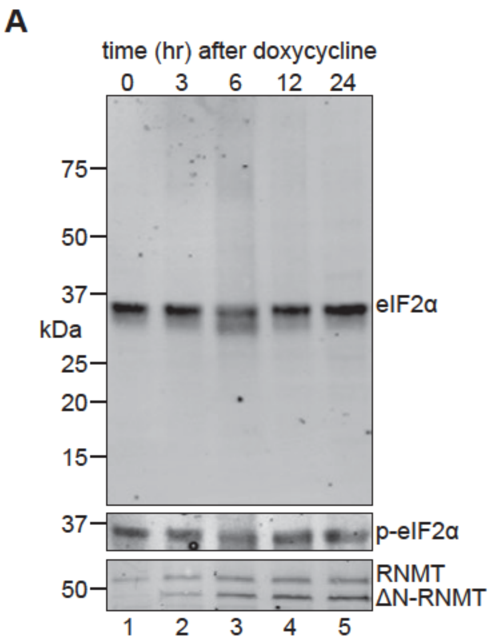

Figure S3 (Related to Table 2)

ΔN-RNMT induction does not impact the integrated stress response or global translation. A. Doxycycline (1 μg/ml) was added at time 0 to cells carrying the tetracycline-inducible ΔN-RNMT transgene. Cytoplasmic extracts recovered at the indicated times were analyzed by Western blotting with antibodies to eIF2α (Proteintech, 1170-1-AP), phospho-eIF2α (Cell Signaling, D9G8), and RNMT (bottom panel). **B.** The global impact of ΔN-RNMT on translation was determined by pulse labeling with puromycin as described in Schmidt, E. K., et al. (Nat Methods 6, 275-277, 2009). Triplicate cultures were incubated for 24 hr without (lanes 1-6) or with (lanes 7-9) doxycycline. They were then treated for 10 min with DMSO (-puromycin) or 10 μg/ml puromycin, washed twice with pre-warmed medium and incubated for an additional 50 min prior to harvest. Puromycin incorporation was determined by Western blotting with anti-puromycin monoclonal antibody (12D10, Millipore Sigma MABE343).

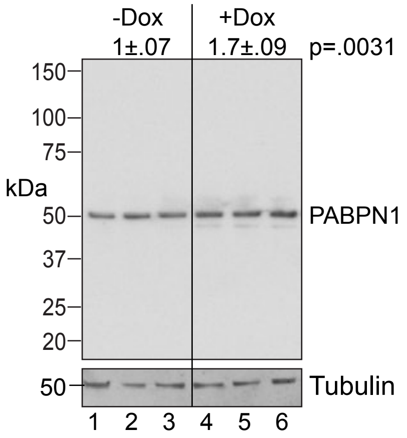

Figure S4 (related to Figure 5)

PABPN1 is elevated in ΔN-RNMT expressing cells. Cytoplasmic extracts were prepared as in Figure S3 from triplicate cultures of U2OS cells carrying the ΔN-RNMT transgene that were incubated for 24 hr in the absence (-Dox) or presence (+Dox) of 1 μg/ml doxycycline. Equal amounts of protein were loaded onto an SDS-PAGE and Western blotting was performed with mouse antibodies to PABPN1 (Proteintech 66807-1-Ig) or α-tubulin (Sigma T6199). Western blots were visualized using anti-mouse Alexa Fluor 594 (Thermo Fisher A11005). PABPN1 normalized to α-tubulin is shown as the mean ± standard deviation. Statistical significance was determined by Student T-test in GraphPad.
